# Real-World Evidence Gathering in Oncology: The Need for a Biomedical Big Data Insight-Providing Federated Network

**DOI:** 10.3389/fmed.2019.00043

**Published:** 2019-03-08

**Authors:** Tine Geldof, Isabelle Huys, Walter Van Dyck

**Affiliations:** ^1^Healthcare Management Centre, Vlerick Business School, Ghent, Belgium; ^2^Department of Pharmaceutical and Pharmacological Sciences, Research Centre for Pharmaceutical Care and Pharmaco-economics, KU Leuven, Leuven, Belgium

**Keywords:** real-world data, real-world evidence, oncology, data exploring, federated network, common data model

## Abstract

Moving toward new adaptive pathways for the development and access to innovative medicines implies that real-world data (RWD) collected throughout the medicinal product life cycle is becoming increasingly important. Big data analytics on RWD can obtain new and powerful insights into medicines' effectiveness. However, the healthcare ecosystem still faces many sector-specific challenges that hamper the use of big data analytics delivering real world evidence (RWE). We distinguish between exploratory (ExTE) and hypotheses-evaluating (HETE) studies testing treatment effectiveness in the real world. From our experience and in the context of the four V's of data management, we show that to get meaningful results data Variety and Veracity are needed regardless of the type of study conducted. More so, for ExTE studies high data Volume is needed while for HETE studies high Velocity becomes essential. Next, we highlight what are needed within the biomedical big data ecosystem, being: (a) international data reusability; (b) real-time RWD processing information systems; and (c) longitudinal RWD. Finally, in an effort to manage the four V's whilst respecting patient privacy laws we argue for the development of an underlying federated RWD infrastructure on a common data model, capable of bringing the centrally-conducted big data analysis to the de-centrally kept biomedical data.

## Background

Real-world data (RWD) collected throughout the medicinal product life cycle will enable more flexible forms of access to innovative medicines, as well as adaptive pathways for their development (1), for example, by providing relative treatment effectiveness evidence during the intensive regulatory processes related to market indication approval, pricing, and reimbursement (2). Moreover, real-world evidence (RWE) captured in clinical practice provides information that complements the internally valid evidence on safety and efficacy generated by randomized controlled clinical trials. Especially so for precision medicines and immune therapies characterized by narrow patient populations, as typically found in the oncology domain. This additional complementary RWE can be used to optimize treatment pathways and resource allocation and ultimately to support outcome-based pricing and reimbursement decisions within early-access managed entry agreements (1–3).

Two types of RWD studies can provide important insights: Hypothesis Evaluating Treatment Effectiveness (HETE) studies, which test a specific hypothesis in a specific population using research-driven data, and Exploratory Treatment Effectiveness (ExTE) studies, or data-driven research that seeks to learn more about possible treatment effectiveness (4). By generating RWE using such ExTE studies, big data analytics can provide new and powerful insights into the effectiveness and performance of products among their specific real-world population and healthcare systems (5). This is why big data in health clinical research is recognized for its “transformative potential” in regard to patients' health, because it tracks not only drug safety and usage but also drug effectiveness (6).

For big data analysis to draw meaningful conclusions, it should consider data from the perspective of the four V's: Volume, Variety, Veracity, and Velocity (7, 8). Hence, the analysis should consider a large *volume* of data tapped from different sources representing the largest possible *variety* of data types offering the most complete picture of the real world, while demonstrating *veracity*, that is, providing an accurate picture of the real world, and becoming available at the highest practical *velocity*, so that data scientists can convert data into insights in an ongoing and up-to-date fashion. To offer a workable solution in real-life clinical healthcare practice, the collected RWD should come from large automatically and continuously collected datasets not collected for one specific study but instead systematically captured from different existing data sources (9).

**Figure 1:**
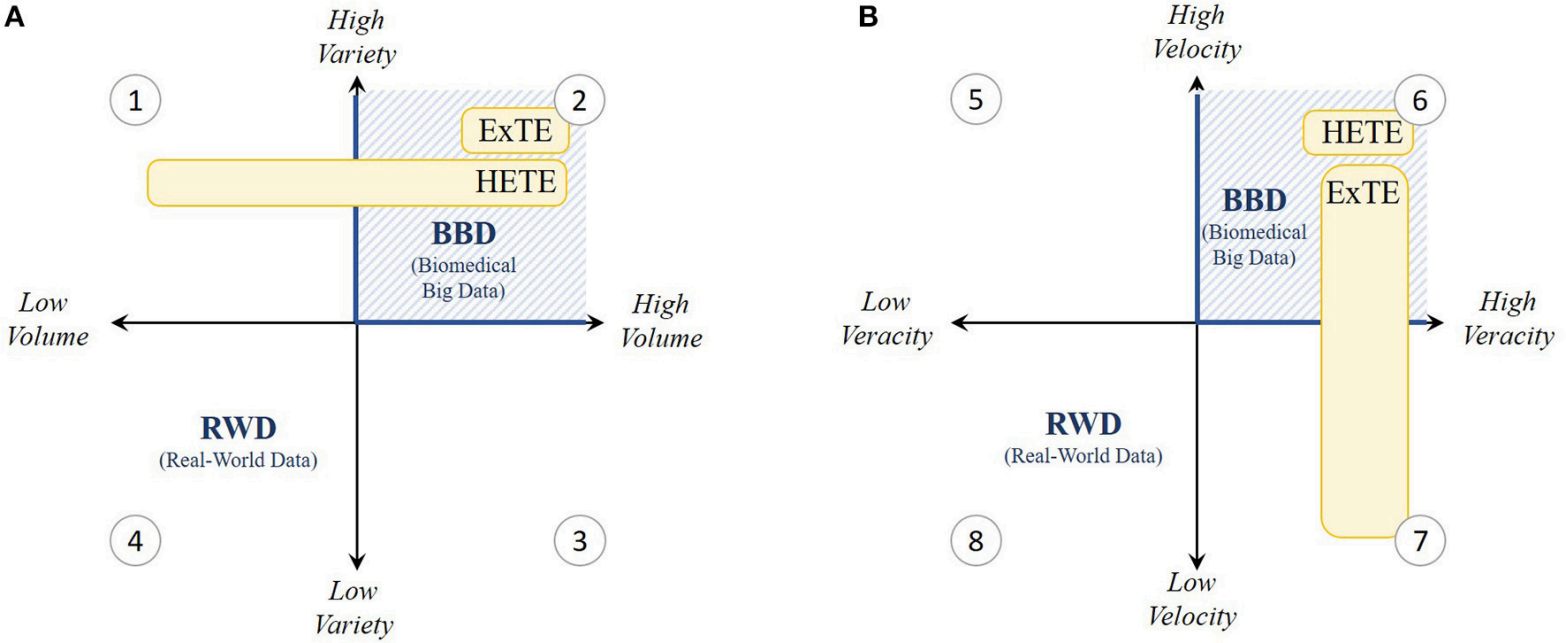
Grid visualization of the present real-world data (RWD) setting within the 4 V's; **(A)** volume and variety and **(B)** velocity and veracity. As indicated in yellow, V's especially important for Hypothesis Evaluating Treatment Effectiveness (HETE) and Exploratory Treatment Effectiveness (ExTE) studies are shown to be variety, velocity and veracity (quadrants 1, 2, and 6) and variety, volume and veracity (quadrants 2, 6, and 7) respectively. Hence, we experience that present RWD settings need to be converted toward biomedical big data (BBD) settings (blue hatched lines) characterized by high V's.

There already exists a large variety of RWD sources that can broadly be categorized as non-interventional studies (pragmatic clinical trials); national-level patient registries (e.g., the Belgian Cancer Registry [BCR]); claims databases (Belgian National Institute for Health and Disability Insurance [NIHDI] and Inter Mutualistic Agency [IMA]); patient surveys and electronic medical records; local hospitals, national, and European biobanks; and hypothesis-driven prospective data sources. However, this decentralized, diverse, and unintegrated nature of the data ecosystem presents problems in the field of data collection and volume, in addition to challenges in data security, exchange, and collaboration between all stakeholders within healthcare (6, 10). These challenges create problems for RWD-based research in oncology, especially in cases involving precision medicines and orphan drugs (for small-patient populations) for exploratory purposes and in cases involving the use of predictive analytical methods. To evolve toward data-driven research and value-based care using biomedical big data, RWD should first and foremost become findable, accessible, interoperable, and reusable (FAIR), the four foundational principles according to Wilkinson et al. (11). As we will show further, for big data to become useful in healthcare, as indicated in Figure 1 RWD settings have to be converted into biomedical big data settings, where managing data volume, variety, and velocity is vital. Therefore, we will reflect on some remaining RWE challenges in the field of oncology from our own ExTE study experience, framed within the four V's of biomedical big data. Next, we discuss three key action points needed within the oncology data ecosystem. Finally, in an effort to manage the four V's whilst respecting patient privacy laws, we argue for the development of an underlying federated RWD infrastructure, capable of bringing the centrally-conducted big data analysis to the de-centrally kept data.

## The Four V's

### Large Volume and Variety

Although identifying appropriate data sources relevant to address a particular research question (i.e., research-driven data selection) is a challenge of its own, the selection of a single retrospective data source is often insufficient for adequate research on ExTE studies because of the limited amount of information collected (variety) and number of patients covered (volume). Also notable are the few sources that can provide comprehensive longitudinal follow-up information (variety), which is a necessity for assessing innovative cancer treatments that have the opportunity to significantly prolong patient lives, while showing benefits in tumor and disease regression and quality of life.

In our exploratory data analysis experience of studying precision-oncology drugs for the treatment of metastatic colorectal cancer patients (diagnosed between 2006 and 2013), problems arose because the BCR does not provide any long-term follow-up (i.e., tumor response) or biomarker information (i.e., molecular targets associated with the cancer). For example, many patients from the BCR had censored survival time, making the study results highly uncertain: patients treated with the precision medicines bevacizumab, cetuximab, panitumumab, or aflibercept resulted in 11–52% censored survival outcomes. On a related note, the lack of biomarker information can lead to biased outcomes. This is caused by the problem of spuriousness, meaning that discovered correlations can be misinterpreted and result in wrong conclusions about causation because of low data variety, which makes the confirmation of associations and the disentanglement of causation from association particularly challenging. Now, the need for sufficient data variety is important in both HETE and ExTE studies (Figure 1A).

Additionally, the collection of metastatic colorectal cancer patients treated with bevacizumab, cetuximab, panitumumab, or aflibercept resulted in a mere 30–2700 eligible patients in Belgium. Although this would be sufficient to conduct HETE studies, these figures are too small to be relevant for exploratory big data analytic studies (5). Insufficient amounts of data can lead to making wrong and ungeneralizable claims because of problems such as “overfitting” (i.e., having a model that fits retrospective data well, but fails prospectively because of failure to enforce proper statistical treatment of data). This problem increases with the ratio of number of parameters adjusted or hypotheses tested to the data volume (number of patients). Especially for colorectal cancer cases, high disease complexity, treatment diversity (including precision drugs), patient (genetic) heterogeneity, and censored effectiveness compound such problems. This means for our case that current country-level sources of RWD in oncology do not offer nor sufficient variety nor sufficient volume to mitigate the problems of spuriousness and overfitting. This is because such datasets should normally exceed a few tera- or even petabytes for big data analytics to be robust and to add value. Especially for rare diseases or medicinal products, such as precision medicines, targeting specific individuals with genetic abnormalities, it is difficult to collect sufficient RWD in one country. Thus, while a low variety is primarily a problem for HETE studies, both aspects of variety and volume are vital in ExTE studies (Figure 1A).

This lack of volume and variety creates the need for a more “hybrid approach,” that is, an approach linking multiple databases capturing different information and covering different countries, thereby allowing analysis across multiple data silos. Based on the previously cited example in Belgium, this hybrid approach is possible today for cancer research using data from the BCR and IMA, both of which are provided with appropriate tools for collection, selection, and merging. In this case, linking other sources such as biobanks could couple genomic data to the phenotypical and transactional BCR-IMA data. Further, to increase available data volume and variety, cross-network, and cross-border collaboration is important.

However, merging databases remains a problematic task because of (i) different data structures and representations across sources and (ii) variable provisions in national legislations for privacy laws covering research practices across countries, despite the General Data Protection Regulation (GDPR), as well as variable implementation of these provisions. This means that current retrospective data sources in Belgium do not meet the “findability” and “interoperability” aspects of the FAIR data principles because most RWD does not have a globally unique and persistent patient identifier (“Findability”) and cannot be merged from different heterogeneous sources based on these identifiers, contents, and representations (“Interoperability”) (11).

### High Velocity and Veracity

Whereas personal data are constantly collected from a wealth of non-traditional sources such as wearables, the collection, storage, and dissemination of personal RWD in health are strongly regulated, especially since the GDPR became enforceable from 25 May 2018. However, even before this, data processing faced many challenges with respect to velocity and veracity. This is especially true for research on precision medicines and orphan diseases where datasets become so small that anonymized data becomes subject to the risk of re-identification. Additionally, particular aspects of the GDPR still remain to be regulated at the member state level, even for international systems. Access for external researchers is therefore often constrained through local rules or privacy laws, especially if global centralized data sources are set up. This hampers the linkage between databases, in some cases at the expense of data quality, while resulting in inevitably lengthy access application processes that are sometimes exacerbated by a lack of incentives for data providers and privacy commissions. Given the time constraints for data collection and research within adaptive outcome-based procedures, fast data processing becomes highly important for HETE studies (Figure 1B). As an example, in Belgium, additional evidence generation for managed entry agreements needs to start within 1 month after contract entry and needs to be submitted to the payer agency after a deadline of only 3 years. However, in our ExTE case cited above, permission to transfer the data to a highly secure academic-hosted server was granted after an application process of approximately 1 year with survival information having a time lag of 2 years between the last patient's status update and actual data supply. Additionally, for both retrospective and prospective sources, RWD are processed only after data collection ends and can rarely be extended to newly diagnosed patients and patient information, thereby limiting the relevance of such data for managed entry agreements.

If no access to adequate data source(s) can be gained to generate specific regulatory-acceptable evidence within HETE studies, new prospective registries–which can be characterized as research-driven and product-specific RWD–need to be set up for collecting appropriate data and providing the evidence needed. This is often the case for many industry-specific research questions raised either by the difficulty of selecting appropriate sources or by constraints in data access. Unfortunately, such studies are very lengthy and costly processes in which data collected during prospective studies are not reusable for other HETE or ExTE studies (e.g., for exploring comparative or combined treatment effects) as opposed to disease-specific registry-based RWD, which have broader potential for data reuse. Thus, although prospective registries are valid for the specific HETE study they were set up for, prospective studies themselves often come at the expense of data veracity, referring to biases, inconsistencies, incompleteness, and possible abnormalities in the data, which jeopardizes study credibility.

On a related note, high data veracity is not only necessary for HETE studies, but is also accepted to be of vital importance for ExTE studies at face value (Figure 1B), because the insights and generated evidence obtained from big data analytics are only as high-quality as the data being analyzed. However, as long as no adequate models are in place to connect different RWD sources having sufficient overlap in information content (i.e., information on the same or similar patient populations), high veracity is difficult to ensure (12). Therefore, a common data model (CDM) is needed that standardizes both data structure and semantics, which improves the interoperability and hence veracity across data sources and countries.

In sum, although different data providers have their own interpretations of FAIR data principles (which are not necessarily aligned with each other), we believe that retrospective RWD for both HETE and ExTE studies do not meet the FAIR criteria. In our example, the necessary IT solutions for smooth data collection and accessibility are present in the Belgian BCR-IMA hybrid database but lack continuous processing and global merging capabilities. Hence, these retrospective RWD do not satisfy the “findability,” “accessibility,” and “interoperability” conditions of the FAIR data principles (11). Because there is no global unique identifier, models are necessary that enhance the use of a common encryption method, enabling fast, and simple linkage between different data sources. An example of this is the use of unique encryption strategies based on social security number, which is currently not the standard method in Belgium. Second, a CDM should urge different sources to transform their content into a common, broadly applicable structure and representation in terms of terminologies (following FAIR principles) for making databases interoperable.

## Need for a Federated Network

**Figure 2:**
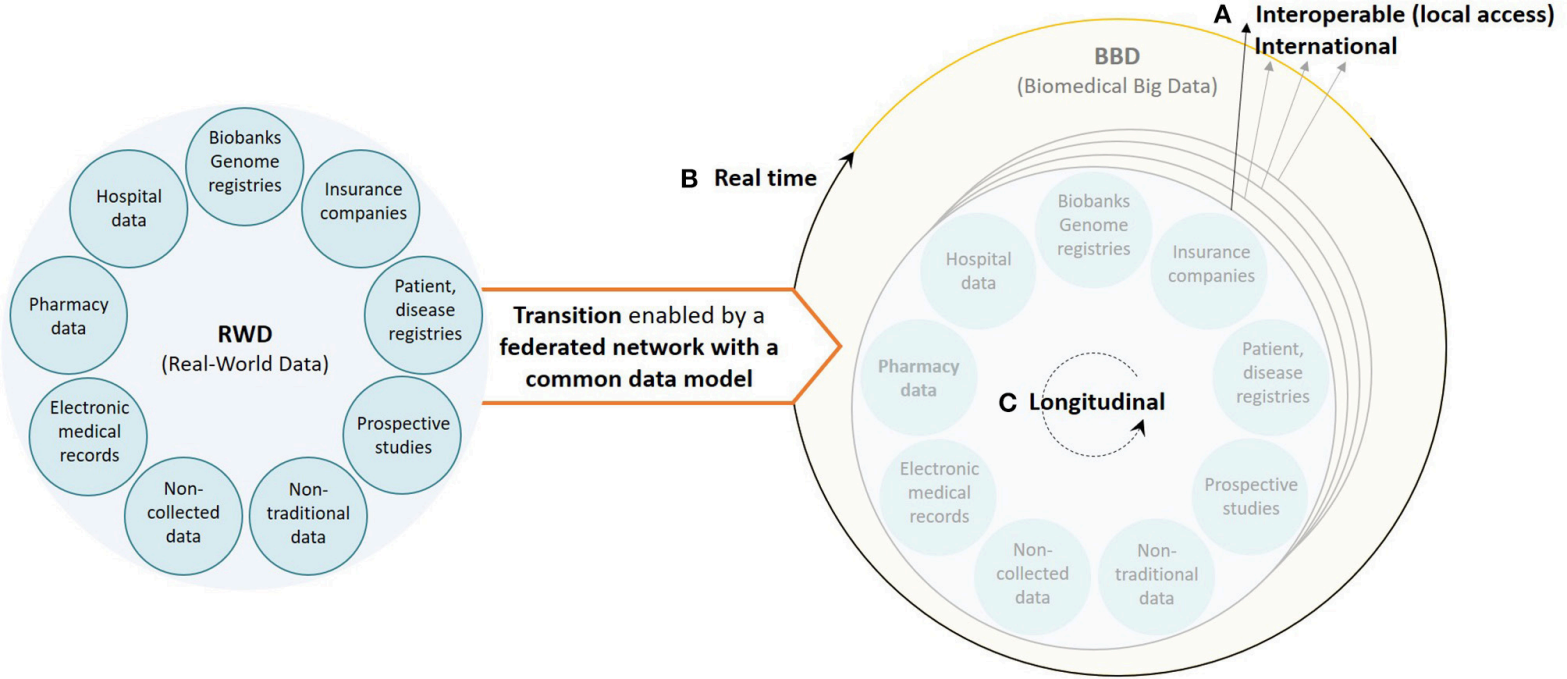
Visualization of the current healthcare data ecosystem (left) and the underlying, federated, data provider infrastructure, based on a common data model, needed to ensure a smooth transition from real-world data to real biomedical big data (right) characterized by **(A)** interoperable databases with international data reusability (high volume and variety), **(B)** real-time RWD processing information systems (velocity), and **(C)** longitudinal data (variety).

It is clear that new actions are needed to transform retrospective (and prospective) RWD to FAIR data to support data-driven research, specifically in environments where patient populations become smaller. For this to happen there are still three important aspects that need attention (summarized in Figure 2) based on our own experience: (i) the databases should aim for reusability on an international scale, accessible through a CDM, facilitating both HETE and ExTE; (ii) the information systems should be able to analyze RWD on an ongoing (as opposed to one shot) basis to support value-based healthcare, facilitating outcome-based managed entry agreements based on HETE studies; and (iii) the databases should become longitudinally oriented to investigate long-term treatment effects for the most innovative medicines for both HETE and ExTE studies. To fulfill these three criteria, we believe that an underlying, federated, data provider infrastructure is vital to stimulate collaboration and data science-based innovation on, at least, a European scale.

Some recent initiatives implementing the needed change are worth to be noted. First, to enable federated discovery of disease-causing mutations in a privacy-preserving fashion, a tool is needed to increase data volume and variety for ExTE genome studies. Next-Generation Sequencing (NGS)-Logistics, the clinical genome resource of ELIXIR Belgium (13) is an example of such a tool.

Second, the US Sentinel CDM enables standardization on a national level. As a centralized system, Sentinel is primarily designed to address regulatory HETE needs (14). However, in Europe, data providers are gradually transforming their datasets conform with the Observational Medical Outcomes Partnership (OMOP) CDM (15), globally supported by the Observational Health Data Sciences and Informatics (OHDSI) research community, making the Sentinel CDM less relevant at a European scale.

Third, the European public-private Innovative Medicines Initiative started EHDEN in 2018, the European Health Data and Evidence Network to create a sustainable and trustworthy European ecosystem (16). The network is based on a federated data platform using an OMOP CDM and International Consortium for Health Outcomes Measurements (ICHOM) outcome standards (a standard patient-centered outcomes set). The platform will leverage vast data volumes and diverse longitudinal patient-level data to support fast and highly reproducible HETE and ExTE studies while respecting (local) privacy regulations, local data provenance, and governance with respect to GDPR. Moreover, the initiative is allied to the open science OHDSI collaboration in real-world, observational research (16).

The underlying data infrastructure for efficient analysis of RWD from a federated network such as EHDEN could rely on a data infrastructure platform provided by NGS-Logistics. Such a tool supports the principle of bringing the “analysis to the data” instead of the “data to the analysis,” the latter typically found in a centralized environment. Key features are that (i) patient data remains under the control of the original data controllers at all times, never leaving their servers, (ii) only queries returning aggregate non-personal results are allowed, and (iii) users are managed via a strict access control system. Keeping the data local in the regional databases allows for respecting national privacy GDPR regulations, local data provenance, and governance.

## Challenges and Considerations

While it is clear that large-scale efforts are needed, some challenges remain inevitable. For example, CDMs relying on a single encryption method can jeopardize the risk of re-identification, because pseudonymization may break down once enough data is linked together. Additionally, federation of de-centrally kept data is practically and legally more complex than data centralization. However, we believe that changing healthcare for the first time into a data-driven sector, creating a shift in data culture and aligning all data providers, is the biggest challenge so far. Overcoming the existing inertia and stimulating industry- and sector-wide collaboration is thus needed to create longitudinal relationships and a sustainable, thriving research community.

Enough incentives will need to be in place to convince data providers to invest in the structuring of their data content and infrastructure to implement this CDM in a federated structure. Translating the value of such data into meaningful economic incentives rewarding the hard work and expertise of clinicians and the labor of data curators is key to moving the collection of high-quality clinical data away from being perceived only as a cost center. Moreover, these incentives will need to be implemented across Europe to enable the required interoperability. Public-private partnerships involving clinicians, public health authorities, health technology companies, and the pharmaceutical industry are probably the answer to co-create sustainable economic value and high quality biomedical big data insights, which clearly reflects the value and relevance for both researcher and data provider.

## Conclusions

Medical researchers are familiar with the opportunities RWD have in a medicinal product's life cycle in regard to improving healthcare practice. This can be seen in the data ecosystem that is trying to leverage improved and unique RWD sources, resulting in biomedical big data being captured everywhere. However, although a large amount of biomedical data is now starting to be captured, many challenges need to be overcome to facilitate appropriate RWD research. As we have shown above, ensuring both high data variety and veracity are important for any (big data analytics) study. More so, high data velocity and volume are essential for respectively HETE and ExTE studies. Here, we formulated three aspects that need attention in the domain of oncology to enable a digital revolution: (i) the transition to international data reusability, (ii) the use of real-time RWD processing information systems, and (iii) the capture of longitudinal RWD.

Recent national initiatives are taking the first steps toward FAIR RWD across Europe. However, we found that an underlying, federated, data provider infrastructure operating on a CDM is key to enable biomedical big data-driven research on the required international scale. EHDEN is the first federated data platform utilizing global outcome standards (ICHOM), achieving interoperability and harmonization (OMOP), and obeying FAIR principles. Efficient analysis of RWD from EHDEN can be realized by a data infrastructure platform such as NGS-Logistics. These projects will definitely provide many future opportunities for RWD in research. However, to stimulate national data providers to invest in the implementation of this CDM following a federated approach, a shift in data-culture is needed. Therefore, strong public-private business cases will have to be developed based on interdisciplinary collaboration ensuring the co-creation of insights. This represents not just a technical but also a national health policy priority-setting problem to be solved, which does not make things easier.

## Author Contributions

The exploration studies mentioned in this manuscript were conducted by TG. TG and WV made substantial contributions to the writing of the manuscript. All authors contributed to the manuscript revision and approved the final version for publication.

### Conflict of Interest Statement

The authors declare that the research was conducted in the absence of any commercial or financial relationships that could be construed as a potential conflict of interest.
